# The long road for vaccine development with difficulties and hopes

**DOI:** 10.1080/22221751.2024.2396886

**Published:** 2024-08-23

**Authors:** Qi Ge, Pan Chen, Yan Cheng, Yonghong Xiao

**Affiliations:** a State Key Laboratory for Diagnosis and Treatment of Infectious Diseases, National Clinical Research Center for Infectious Diseases, Collaborative Innovation Center for Diagnosis and Treatment of Infectious Diseases, the First Affiliated Hospital, Zhejiang University School of Medicine, Hangzhou, People’s Republic of China; b Peking Union Medical College & Institute of Pathogen Biology, Chinese Academy of Medical Sciences & Research Units of Infectious Disease and Microecology, Chinese Academy of Medical Sciences, Beijing, People’s Republic of China; c Department of Structure and Morphology, Jinan Microecological Biomedicine Shandong Laboratory, Jinan, People’s Republic of China

The R21/Matrix-M malaria vaccine (which has an overall protection rate of 75%) was listed as a pre-qualified vaccine by the World Health Organization (WHO) in December 2023; as such, it is the second malaria vaccine to be authenticated by WHO after pre-qualification of the RTS, S/AS01 (overall protection rate = 55.1%, both vaccines are designed to target the circumsporozoite protein (CSP). R21 features a higher density of CSP epitopes on its surface, requiring a lower dosage than RTS,S. However, without direct comparative trials, it remains unclear which vaccine is superior.) vaccine in July 2022 [1–3]. Attempts to develop an effective vaccine for malaria, one of the most severe parasitic diseases in human history, have been ongoing since the 1960s; however, the huge and complicated plasmodium genome, the intricate life cycle of the parasite, and the diverse human immune responses that it triggers, have made this task extremely difficult.

However, the fact that the WHO has recommended two malaria vaccines in consecutive years suggests significant progress in the field. This accomplishment not only suggests that humanity has gained the upper hand in the fight against malaria, but also signifies a breakthrough in vaccine development, demonstrating the successful transition from theoretical research to practical applications in combating diseases caused by eukaryotic microbes.

The year 2023 proved to be a bumper year for vaccine development. Apart from the new malaria vaccine, we have seen a breakthrough with respect to a respiratory syncytial virus (RSV) vaccine. RSV takes a heavy toll on the health of young children and the elderly worldwide. RSV vaccines have been around for more than 50 years; however, because the viral F protein (the target antigen) undergoes extensive conformational changes before and after mediating fusion, it has been very difficult to develop a vaccine with high efficacy. Recently, the three-dimensional pre-fusion structure of the F protein was elucidated, and scientists could make use of the achievement to design a recombinant vaccine targeting the stable pre-F conformation. This resulted in a significant breakthrough in the field of RSV vaccine research and development. In 2023, two consecutive RSV vaccines, named Arexvy, Abrysvo, with overall protection rates of 82.6% and 84.4%, respectively, were approved by the FDA. Both vaccines are designed to target the RSV-F protein, yet clinical studies of Abrysvo indicate that it can also prevent neonatal RSV infections when administered during pregnancy. These vaccines will help to prevent and control RSV infection and provide protection for high-risk groups such as people aged over 60 years and newborns [4–7].

Also noteworthy is the major progress in mRNA vaccine development. The phase III clinical trial (conducted in 2023) of a personalized mRNA vaccine (mRNA-4157/V940) plus a monoclonal antibody (pembrolizumab) as a combined treatment for high-risk melanoma is a milestone on the road to finding a treatment for this disease [8]. Additionally, the Nobel Prize in Physiology or Medicine was awarded to biochemist Katalin Karikó and immunologist Drew Weissman for their pioneering work that paved the way for developing mRNA vaccines against COVID-19. The favourable safety profiles, high clinical efficacy, potential for rapid development and low production costs make mRNA vaccines a promising alternative to more “traditional” vaccines. Prior to emergence of COVID-19, mRNA technology was used mainly to develop novel therapeutic agents that target malignant tumours. The COVID-19 pandemic created an urgent need for the rapid development of vaccines that are easy to scale up, manufacture, and demonstrate both safety and immunological efficacy. The upside of the pandemic is that it has propelled advancement of mRNA vaccine technology and increased public awareness and acceptance of mRNA vaccines. Thus, mRNA vaccines became a powerful weapon in the fight against COVID-19, ushering in a new era of vaccine development. Certainly, many vaccine achievements in recent years were not mentioned above, we have listed the vaccines that have made significant breakthroughs since 2020 (Table 1).

**Table 1.** Vaccines with breakthrough developments available on the market since 2020.

Type	Name	Pathogen	Advantages	Disadvantages	Time
nucleic acid vaccine	mRNA-1273 BNT162b2	SARS-CoV-2 SARS-CoV-2	Strong immunogenicity and high specificity Rapid development: Designed based on genetic sequences, without the need to culture pathogens. Safety: Does not involve the use of pathogenic organisms in the vaccine formulation. Personalized formulation: Allows for reengineering or optimization of vaccines for specific pathogen strains or patient-specific cancer cells by modifying the nucleic acid sequences. DNA vaccines are easy to store and transport. Facilitates large-scale manufacturing.	High development complexity and limited dissemination of production technology. The efficacy of the immune response may diminish over time or due to pathogen mutation. There are potential safety concerns regarding the integration of DNA from DNA vaccines into the host genome. mRNA vaccines require stringent storage and transportation conditions due to the susceptibility of the vaccine components to degradation, necessitating cold-chain logistics.	2020 2020
	mRNA-1345	RSV			2024
	ZyCoV-D (DNA vaccine)	SARS-CoV-2			2021
recombinant protein vaccine	RTS, S/AS01 R21/Matrix-M	plasmodium	High specificity Safety: Does not involve the use of pathogenic organisms in the vaccine formulation and eliminates the risk of genetic integration into the host genome. Convenient storage and transportation. Mature technology	Limited flexibility: Immunogenic efficacy may decline in response to pathogen mutations. Requires adjuvant addition or multiple doses for optimal effectiveness. Complex manufacturing process. Immunogenicity is influenced by the expression system utilized.	2022 2023 2023 2023 2022
	Arexvy Abrysvo Heplisav-B	RSV RSV HBV			
attenuated live vaccine	TAK-003	dengue virus	Strong immunogenicity with long-lasting immune effects. Broad immune coverage across multiple pathogen components. Requires fewer doses for effective immunization and o adjuvant needed. Contributes to partial herd immunity.	Lower safety profile: Potential for mild infection symptoms, risk of virulence reversion, possible environmental transmission of the pathogen, and unsuitability for immunocompromised individuals and pregnant women. High storage and transportation requirements.	2024
	Ixchiq	chikungunya virus			2023

Vaccines are one of the most outstanding achievements in the fields of medicine and public health. Since Edward Jenner developed the cowpox vaccine 200 years ago, every step on the road to vaccine development has improved human health significantly. The cowpox vaccine provided a glimmer of hope in a dark age, giving people the optimism to fight, and even eliminate, smallpox, the most dreaded infectious disease in human history. Development of live attenuated and inactivated vaccines 140 years ago marked the dawn of a new era. Since then, many vaccines have been developed to combat various diseases. Development of subunit vaccines 50 years ago enabled cost reductions and improved efficiency of production; thus, mass vaccination programmes could be initiated to limit outbreaks of many diseases. Since the turn of the century, vaccine development has accelerated alongside technological advancements. Successful development of a vaccine against human papilloma virus (HPV) is another milestone in cancer prevention, while emergence of reverse vaccinology has expanded our understanding of vaccine development. Many previously insurmountable problems in vaccine development, which was initially guided by the classical Pasteurian method of “isolation, inactivation, and injection” have finally been resolved, thereby expanding the scope of vaccine application [9]. As mentioned above, development of mRNA vaccines in response to the COVID-19 pandemic increased the speed of vaccine development markedly, providing us with powerful tools to combat emerging infectious diseases quickly and effectively. At the same time, the customizable characteristics of mRNA vaccines have opened up new avenues for development of anti-cancer vaccines.

Despite their tremendous impact on controlling and eradicating a wide range of infectious diseases, demand for vaccines against existing and emerging diseases is enormous. Some diseases for which successful vaccines have yet to be developed, e.g. AIDS, continue to place enormous pressure on global public health systems. Other diseases for which vaccines are already in use, e.g. tuberculosis and influenza, require more effective products. Changes in the pathogens themselves mean that original vaccines become less efficient in that are only able to target specific populations for short periods of time. Emergence of new infectious diseases such as COVID-19 has highlighted the importance of vaccine research and development. Therapeutic anti-cancer vaccines are also highly anticipated. The complexity and high variability of pathogens, particularly in the context of pathogenesis and immune escape mechanisms, along with a lack of theoretical guidance, mean that developing and optimizing vaccines is challenging. For instance, during the early stages of dengue vaccine research, we did not understand cross-reactions among the four serotypes of the dengue virus, or the phenomenon of antibody-dependent enhancement. This led to difficulties in selecting appropriate vaccine target antigens. Once ADE was elucidated, it became clear that an effective dengue vaccine must induce a broad neutralizing antibody response against all four serotypes. Moreover, pathogens showing high levels of phenotypic variation make vaccine development complicated because the vaccines need to target multiple variants simultaneously. For example, the high variability of HIV means that vaccines must induce production of broadly neutralizing antibodies (bnAbs) that recognize the most prevalent strains of the virus [10]. Insights into the immune mechanisms of individuals who naturally develop bnAbs are crucial for vaccine design. Variability in immune responses among populations can lead to poor vaccine efficacy in specific groups, such as the generally lower protection offered by influenza vaccines to the elderly. There's a need to strategically utilize existing vaccine platforms, such as avoiding live-attenuated vaccines, selecting appropriate adjuvants and administration methods, and employing passive immunization to ensure safe and effective immunity in individuals with weaker immune systems. Despite overcoming the aforementioned issues, vaccine development still faces numerous challenges in clinical trials. Differences between human and animal models can raise safety concerns. Effective evaluation in humans often requires long-term monitoring and extensive data collection. Moreover, conducting experiments under varying legal and ethical constraints worldwide makes experimental design, data analysis, and interpretation formidable challenges, leading to many vaccines failing during clinical trials.

In addition, the cost, production, storage, and transportation of vaccines vary across global regions, and different countries have different vaccination policies. In addition, public confidence in vaccines varies, placing constraints on application and efficacy. Particularly in low-resource settings, the lack of technical funding, inadequate infrastructure, and vaccine hesitancy among the public create significant barriers to vaccine development. This situation requires local governments to focus on specific local needs and actively seek external collaboration.

Taken together, these factors make vaccine development challenging, and the road to future vaccine research and development is full of thorns. However, it is the desire to overcome these difficulties that has driven exploration of new technologies, as well as the deepening of established theories, leading vaccine research and development in promising new directions.

In developing the smallpox vaccine, Jenner was unaware that the disease was caused by a virus. Similarly, Pasteur, while establishing the fundamental principles of vaccinology, had no knowledge of immunological mechanisms [11]. Although vaccine development has long been rooted in “empiricism,” recent advances in biological sciences have undeniably accelerated vaccine innovation. The evolution of structural biology now allows scientists to observe interactions between pathogens and host molecules at the atomic level through techniques like X-ray crystallography and cryo-electron microscopy. Additionally, advancements in bioinformatics have introduced reverse vaccinology, a novel approach to vaccine design. Traditional biology has amassed a wealth of knowledge on immunology and host–pathogen interactions, laying a solid foundation for the continual optimization of vaccine design.

The progress in vaccine development platforms and technologies has brought about revolutionary changes in modern vaccinology. Traditional vaccines, primarily based on live attenuated, inactivated, and subunit vaccines, have been complemented by newer technologies like DNA vaccines, RNA vaccines, and viral vector vaccines. These innovations have addressed some of the safety, immunogenicity, and production efficiency issues associated with traditional vaccines, enabling a rapid response to emerging infectious diseases. A prime example is the Pfizer-BioNTech mRNA COVID-19 vaccine, which was developed in just a few months and successfully administered on a global scale, showcasing the rapid adaptability and efficacy of these new technologies.

Advancements in delivery systems have also been a key driver of recent progress in the vaccine field. Integrating immunogen design with material science and engineering techniques is becoming a future trend in various vaccine platforms. Nanoparticles (NPs) and lipid nanoparticles (LNPs) have resolved issues such as the differing biodistribution and half-life of antigens and adjuvants in the body, which previously led to reduced vaccine efficacy and non-specific inflammation [12]. The progress in material science has enabled the regulation of antigen degradation rates within the body, allowing for more sustained immunogen delivery. Specially designed carriers, based on expanding new materials and immunological knowledge, not only enhance control over in vivo delivery but also potentially direct immune responses along specific pathways of cell differentiation and expansion.

Furthermore, the COVID-19 pandemic sparked global discussions on vaccine approval processes, ethical considerations, and public acceptance. During the pandemic, governments and regulatory agencies accelerated vaccine approval through Emergency Use Authorization (EUA). In the United States, the FDA's EUA for COVID-19 vaccines significantly shortened the time to market. This shift not only saved millions of lives but also prompted a reevaluation and adjustment of global vaccine approval systems. Simultaneously, the “vaccine campaign” during the pandemic significantly increased public awareness and acceptance of vaccines. As vaccination became a critical strategy to combat the pandemic, public confidence in vaccine safety and efficacy also grew.

Looking back at history and forward to the future, the antigenic components of vaccines have become increasingly simple and specific, evolving from whole pathogens to protein antigens, DNA, and RNA [11]. Personalization and precision will be the focus of future research. By integrating precise antigen epitopes into antigen design, utilizing various vaccine development platforms, and combining these with delivery systems capable of eliciting specific immune responses in targeted areas, we can continue to meet the preventive needs for existing and emerging pathogens. This approach also holds promise for significant breakthroughs in areas such as chronic infections, immune diseases, and cancer.

Interdisciplinary collaboration may be key to future breakthroughs in vaccine research. One emerging area is quantum vaccines [13]. Although still in the theoretical exploration stage, advances in quantum biology could bring a new dimension to vaccine design, making development more precise and efficient. The growth of artificial intelligence also has the potential to make intelligent vaccine development a reality.

In summary, recent years have witnessed significant advancements in the vaccine field across multiple aspects, including fundamental sciences, technological platforms, delivery systems, and sociological considerations. These developments have not only enhanced the efficiency and quality of vaccine research and development but also charted a course for the future of vaccinology. As science and technology continue to evolve, vaccines will advance along the paths of personalization, precision, and interdisciplinary collaboration, making even greater contributions to human health.

## References

[1] WHO. WHO prequalifies a second malaria vaccine, a significant milestone in prevention of the disease; [cited 2023 Dec 21]. Available from: https://www.who.int/zh/news/item/21-12-2023-who-prequalifies-a-second-malaria-vaccine-a-significant-milestone-in-prevention-of-the-disease.

[2] Datoo MM, Dicko A, Tinto H, et al. A phase III randomised controlled trial evaluating the malaria vaccine candidate R21/Matrix-M™ in African children. 2023.

[3] RTS SCTPJNEJoM. A phase 3 trial of RTS, S/AS01 malaria vaccine in African infants. N Engl J Med. 2012;367(24):2284–2295. doi:10.1056/NEJMoa1208394 23136909 PMC10915853

[4] Papi A, Ison MG, Langley JM, et al. Respiratory syncytial virus prefusion F protein vaccine in older adults. N Engl J Med. 2023 Feb 16;388(7):595–608. doi:10.1056/NEJMoa2209604 36791160

[5] Kampmann B, Madhi SA, Munjal I, et al. Bivalent prefusion F vaccine in pregnancy to prevent RSV illness in infants. N Engl J Med. 2023;388(16):1451–1464. doi:10.1056/NEJMoa2216480 37018474

[6] Walsh EE, Pérez Marc G, Zareba AM, et al. Efficacy and safety of a bivalent RSV prefusion F vaccine in older adults. N Engl J Med. 2023;388(16):1465–1477. doi:10.1056/NEJMoa2213836 37018468

[7] Melgar M, Britton A, Roper LE, et al. Use of respiratory syncytial virus vaccines in older adults: recommendations of the advisory committee on immunization practices – United States, 2023. MMWR Morb Mortal Wkly Rep. 2023;72(29):793–801. doi:10.15585/mmwr.mm7229a4 37471262 PMC10360650

[8] Moderna and Merck announce mRNA-4157/V940, an investigational personalized mRNA cancer vaccine, in Combination with KEYTRUDA® (pembrolizumab), met primary efficacy endpoint in phase 2b KEYNOTE-942 trial; [cited 2022 Dec 13]. Available from: https://www.merck.com/news/moderna-and-merck-announce-mrna-4157-v940-an-investigational-personalized-mrna-cancer-vaccine-in-combination-with-keytruda-pembrolizumab-met-primary-efficacy-endpoint-in-phase-2b-keynote-94/.

[9] Rappuoli R, Covacci A. Reverse vaccinology and genomics. Science. 2003 Oct 24;302(5645):602. doi:10.1126/science.1092329 14576423

[10] Paneerselvam N, Khan A, Lawson BR. Broadly neutralizing antibodies targeting HIV: progress and challenges. Clin Immunol. 2023 Dec;257:109809. doi:10.1016/j.clim.2023.109809 37852345 PMC10872707

[11] Fiyouzi T, Reche PA. Vaccine design: an introduction. In: Reche PA, editor. Computational vaccine design. New York: Springer US; 2023. p. 1–14. 10.1007/978-1-0716-3239-0_1 37258903

[12] Gebre MS, Brito LA, Tostanoski LH, et al. Novel approaches for vaccine development. Cell. 2021 Mar 18;184(6):1589–1603. doi:10.1016/j.cell.2021.02.030 33740454 PMC8049514

[13] de la Fuente J, Contreras M. Vaccinomics: a future avenue for vaccine development against emerging pathogens. Expert Rev Vaccines. 2021;20(12):1561–1569. doi:10.1080/14760584.2021.1987222 34582295

